# Proliferation of pleural mesothelioma cells is enhanced by the microRNA-197-3p activity

**DOI:** 10.3389/fonc.2026.1807528

**Published:** 2026-06-29

**Authors:** Ilaria Bononi, Giulia Di Mauro, Maria Letizia Tramarin, Valentina Esposito, Giulia Tonnini, Lucia Oton-Gonzalez, Beatrice Dallan, Elena Torreggiani, Fernanda Martini, Mauro Tognon

**Affiliations:** 1 Department of Medical Sciences, University of Ferrara, Ferrara, Italy; 2 Department of Environmental and Prevention Sciences, University of Ferrara, Ferrara, Italy; 3 Center for Studies on Gender Medicine - Department of Medical Sciences, University of Ferrara, Ferrara, Italy

**Keywords:** biomarker, miR-197-3p, pleural mesothelioma, proliferation, target

## Abstract

Human Pleural Mesothelioma (HPM) is an aggressive asbestos-related tumor with limited treatment options and a poor prognosis. MicroRNAs (miRNAs), known to play key roles in the pathogenesis of HPM, have emerged as promising candidates for both diagnostic and therapeutic applications. Among them, miR-197-3p has been previously identified as dysregulated in sera from HPM patients and workers ex-exposed to asbestos fibers. To investigate the functional role of miR-197-3p, loss- and gain-of-function studies were performed in HPM cell lines and human mesothelial cells (HMC) using miR-197-3p-specific antagomiR and mimic. The effects of miR-197-3p modulation on cell proliferation, viability, migration, and apoptosis were evaluated. In addition, bioinformatics analyses were performed to identify potential miR-197-3p target genes, which were subsequently evaluated at both mRNA and protein levels. MiR-197-3p tested significantly upregulated in HPM cells. Its inhibition led to a marked reduction of the HPM cell proliferation, whereas its overexpression in HMC promoted a proliferative phenotype, supporting a potential role in cell growth regulation. Among the predicted targets, TGF-β1 and p120 showed modulation at the mRNA level, although protein-level changes were limited or only partially consistent. These findings suggest that miR-197-3p may contribute to HPM pathogenesis by promoting cell proliferation and influencing critical molecular pathways. However, the underlying molecular mechanisms remain to be fully elucidated, and the interaction with candidate targets, such as TGF-β1 and p120, should be considered putative. Further investigations, including functional and mechanistic validation in more representative experimental models, will be required to clarify the role of miR-197-3p in HPM pathobiology.

## 1 Introduction

Human Pleural Mesothelioma (HPM) is a rare and highly aggressive tumor affecting the pleural cavities, primarily caused by exposure to asbestos, a natural oncogenic mineral (1). The HPM onset may occur after a long latency period, up to 50 years (2). Globally, more than 30,000 HPM cases are reported every year, with an incidence rate of 0.30 cases per 100,000 people (3). Life expectancy after HPM diagnosis is about one year, mainly because current therapeutic options for this tumor are limited with poor outcomes on patient survival, making HPM a fatal disease (4). Despite many Western countries banned asbestos, the HPM incidence is increasing, with a projected peak around 2030 (5), raising significant public health concerns (6). In most cases, HPM arises asymptomatically, leading to late-stage diagnosis and challenging treatment options (1). HPM is classified into three main histotypes, i.e. i) epithelioid, ii) biphasic, and iii) sarcomatoid, each significantly impacting prognosis and treatment strategies (7). Specifically, patients with the HPM epithelioid subtype are more suitable for surgical intervention and exhibit higher survival rates (up to 19 months) compared to the other two histotypes. In contrast, the biphasic and sarcomatoid subtypes are more difficult to cure and patients have significantly lower survival rates, (12 and 4 months, respectively) (8).

The mechanisms underlying the HPM onset remain unclear, but asbestos exposure plays a crucial role (9). Asbestos affects the immune system, recruiting macrophages in the fiber location and activating pro-inflammatory responses (10), leading to persistent inflammation and tissue damage, while promoting the release of proteins, such as HMGB1, facilitating malignant cell transformation (9).

Additionally, genetic mutations and epigenetic alterations contribute to HPM pathogenesis, with significant genes, including BAP1, CDKN2A, and NF2, being often mutated, and various miRNAs implicated in the tumor onset and progression (11). Small non-coding RNA molecules, such as miRNAs, play a crucial role in regulating gene expression (12) and are involved in key cell transformation processes, including tumorigenesis, cancer progression, and drug resistance, across many human tumors, including HPM (1, 2). Moreover, several studies have shown that asbestos exposure modulates miRNA expression, as demonstrated by next-generation sequencing analysis, highlighting the influence of asbestos on miRNA expression profiles (13–17).

These findings and the evidence of the dysregulation of circulating miRNAs in HPM patients’ serum/plasma remark on the important roles of small non-coding RNA in the HPM onset (18). Epigenetic alterations are emerging as key factors in the HPM onset, with miRNAs seen as potential diagnostic markers and therapeutic targets (13–16).

In previous studies we reported a decreased level of circulating miR-197-3p in workers ex-exposed to asbestos (WEA) sera compared to healthy subjects, alongside an inverse correlation between miR-197-3p expression and cumulative asbestos exposure (15). In another report, we showed that circulating miR-197-3p is over-expressed in sera from patients affected by HPM compared to WEA (16).

MiR-197-3p has been documented to exhibit diverse roles in various human cancers, including hepatocellular carcinoma, lung cancer, non-small cell lung cancer (NSCLC), ovarian carcinoma and osteosarcoma, by interacting with different target genes, such as ZIK1, P53 and p120, CYLD, NLK, and EHD2 (19–24). Interestingly, in other contexts—such as cervical, prostate, and breast cancers, fibrosarcoma, and osteosarcoma—miR-197-3p appears to function as a tumor suppressor by targeting ELK1, E2F6, VDAC1, and RAN (25–30).

Herein, we investigated the potential role of miR-197-3p in regulating proliferation-related processes in HPM cells of epithelioid, biphasic, and sarcomatoid histotypes. Normal human mesothelial cells (HMC) were employed as control. Our results suggest that modulation of miR-197-3p influences clonogenic growth, without significantly affecting cell viability, migration or apoptosis, indicating a potential role in proliferation-related pathways.

Overall, our preliminary findings provide insight into the possible involvement of miR-197-3p in HPM biology and support further investigations aimed at clarifying its mechanistic role in this aggressive cancer.

## 2 Method

### 2.1 Cells

HPM cell lines, i.e. (i) the epithelioid MPP89; (ii) the biphasic MSTO-211H and (iii) the sarcomatoid PPM-Mill were from our cell repository, as published before (2, 31). Human mesothelial cells (HMC) were purchased from Celprogen (Clinisciences, Guidonia Montecelio, Italy). All cell cultures were grown in appropriate media in a humidified incubator under sterile conditions at 37 °C with 5% CO_2_ (32, 33). At the confluence, cells were seeded for further analysis or harvested, centrifuged, and cell pellets stored at -80 °C until nucleic acid and protein extractions.

### 2.2 RNA extraction and reverse transcription

Total RNAs, including micro-RNAs, were extracted from cell cultures using the RNeasy Plus Mini Kit (Qiagen, Milan, Italy) following the protocol provided by the manufacturer. The extracted RNA was quantified with NanoDrop 2000 and stored at -80 °C until analysis. For total RNA, complementary first-strand DNA (cDNA) was synthesized from 50 ng of total RNA by reverse transcription using the ImProm-II reverse transcription system (Promega, Milan, Italy) according to the manufacturer’s instructions and stored at -20 °C. MiRNAs were reverse transcribed using the miRCURY LNA RT kit (Qiagen, Milan, Italy) as previously described (15, 16).

### 2.3 Transfection

AntagomiR transfections were conducted on epithelioid, biphasic and sarcomatoid HPM cell lines, whereas mimic transfections were performed on HMC. Briefly, cells were seeded at 3x10^4^ cells/well in a 24-well plate and allowed to adhere for 24 h under standard conditions. The oligonucleotides hsa-miR-197-3p miRCURY LNA miRNA Inhibitor (or antagomiR) and hsa-miR-197-3p miRCURY LNA miRNA Mimic (Qiagen, Milan, Italy) were reconstituted to obtain a 10 µM stock solution according to the manufacturer’s guidelines. Transfection mixes were prepared using 2 μM of mimic/antagomir and the HiPerFect transfection reagent (Qiagen, Milan, Italy) according to the manufacturer’s instructions. After 72 h of incubation under standard growth conditions, cell pellets were collected, and RNA extracted for subsequent analyses. Experiments were conducted in triplicate for each experimental group, i.e.: (i) untransfected control; (ii) mock control, treated only with the transfecting agent and PBS; (iii) transfected with antagomiR or mimic.

### 2.4 Cell colony formation capacity

HPM and HMC cells (1,000 cells/well) were seeded in 24-well plates at 37 °C in a 5% CO_2_ atmosphere overnight. After 24 h, cells were transfected with miR-197-3p siRNAs. At day 10, colonies were washed with PBS, fixed with 100% methanol, and stained with 0.1% crystal violet (12, 34). Plates were scanned and the automated colony counting tool of ImageJ software was used for analysis (34).

### 2.5 Cell viability, cell migration and apoptosis

HPM and HMC cell viability was evaluated by Cell Counting Kit-8 (CCK-8; Merck, cod. 96992) 24, 48, and 72 h after miR-197-3p mimic/antagomiR transfection. Cells were seeded at 5x10^3^ cells/well in a 96-well plate and allowed to adhere for 24 h under standard conditions. Subsequently, fresh complete medium containing 10 µl of CCK-8 was added to each well and incubated for 2 h at 37˚C. Medium containing 10% CCK-8 was used as a control. Cell viability was determined by measuring absorbance at 450 nm with a microplate reader (Sunrise™, Tecan, Milan, Italy).

Cell migration was assessed by wound-healing assay (or scratch test). HPM and HMC cells were seeded at 1.5 x10^4^/well in a 24-well plate. After reaching 90% confluence, a linear scratch was made in the middle of each well using a 200 μl pipette tip to create an artificial wound in the cell monolayer. Each well was washed gently with PBS to remove floating cells. Cells were then transfected with miR-197-3p mimic/antagomiR and imaged under an optical microscope (Nikon, ECLIPSE E200 Instruments Europe BV, Rome, Italy) at time 0 and 24 h. The scratch area was calculated by measuring the gap enclosed by the cells using ImageJ software. The wound healing percentage was calculated according to the following formula: Wound healing percentage = [(At=0 h - At=24 h)] x 100, where At=0 h and At=24 h represent the scratch area measured at time 0 and 24 h after scratching, respectively. The ColonyArea ImageJ plugin was used to analyze the staining intensity.

Apoptosis evaluation was performed by Annexin V staining. HMC and HPM cells were seeded at 1.5 x10^5^/well in a 6-well plate and were transfected with mimic and antagomir for 72 h. Then, cells were trypsin treated and centrifuged at 200 g for 5 min. Cell pellets were washed twice with cold Biolegend’s Cell Staining Buffer (Biolegend, Milan, Italy). Staining was performed according to the manufacturer’s protocol. Briefly, cells were stained with fluorescein isothiocyanate (FITC) Annexin-V and Propidium Iodide (PI) Staining Solution and incubated for 15 min in the dark. Then, Annexin V Binding Buffer was added to each tube. Data was acquired by the BD FACSCanto™ II flow cytometer (BD Biosciences, Milan, Italy) and analyzed using FlowJo version 10 software (FlowJo, BD Biosciences, Milan, Italy).

### 2.6 MiR-197-3p target selection

To identify potential miR-197-3p target genes, several target prediction databases were employed, including miRTarBase (mirtarbase.cuhk.edu.cn), mirDB (mirdb.org), miRDIP (ophid.utoronto.ca/mirDIP/), and TargetScan (targetscan.org). The most promising theoretical target genes were selected based on their predicted down-regulation by miR-197-3p and their potential involvement in mediating its biological effects. Since our findings indicate that miR-197-3p significantly influences cell proliferation, we first analyzed genes with known roles in proliferative pathways (20, 22, 23), such as catenin 1 delta (CTNND1 or p120) and transforming growth factor beta 1 (TGF-β1) (35–40).

### 2.7 Gene expression analysis using droplet digital PCR (ddPCR)

cDNA obtained from retro-transcription was mixed with miRCURY LNA-specific PCR primer set for miRNA detection and EvaGreen supermix 2X (Bio-Rad, Segrate, Italy). The emulsion was obtained using an automatic droplet generator (Bio-Rad, Segrate, Italy) according to the manufacturer’s instructions and the SimpliAmp Thermal Cycler (Thermo Fisher Scientific, Milan, Italy) was used for the PCR amplification. PCR conditions for miR-197-3p analysis included 40 cycles at 95 °C for 30 seconds and 56 °C for 1 minute and three final steps at 4 °C for 5 minutes, 90 °C for 5 minutes to improve dye stabilization, and an indefinite stop at 4 °C. For miR-197-3p target analysis, PCR conditions were similar, with an annealing temperature of 60 °C. Data analysis was performed using the Qx 200 Droplet Reader (Bio-Rad, Segrate, Italy) and Quanta Soft software (Bio-Rad, Segrate, Italy). The levels of miR-197-3p were represented as normalized miRNA expression and data were normalized to the housekeeping U6 (copies x µl^-1^ of miR-197-3p/copies x µl^-1^ of U6) (15, 16).

### 2.8 Protein extraction and expression analysis by western blot

The western blot technique was employed to validate miR-197-3p target gene encoding proteins. Protein extraction was performed in 1X RIPA Buffer containing 10% PhoSTOP, 1% PMSF, and 1X lysis buffer with protease inhibitors. Protein lysate concentration was quantified using the BCA assay according to the manufacturer’s protocol. Samples were prepared in Laemmli sample buffer with 5% β-mercaptoethanol (13) and were separated on 4-15% Mini-PROTEAN TGX precast gels and transferred to Trans-Blot Turbo Mini 0.2 μm Nitrocellulose membrane using the Trans-Blot^®^ Turbo™ Transfer System (Bio-Rad, Segrate, Italy) (41). The membrane was incubated with EveryBlot Blocking Buffer and primary antibodies against p120 (1:500) and TGF-β1 (1:1,000) overnight at 4 °C. β-actin served as a loading control (1:5,000). After washing, the membrane was incubated with the appropriate secondary antibody (1:3,000) conjugated to horseradish peroxidase. Immune complexes were detected by enhanced chemiluminescence using Clarity Western ECL substrate, and images were acquired with a ChemiDoc imaging system. Precision Plus Protein Dual Color Standards were included in each run for molecular weight reference. Protein band quantification was performed using Image Lab Software Target protein expression levels were normalized to β-actin. Molecular weights were indicated in each western blot figure (34).

### 2.9 Statistical analysis

Data were analyzed using Prism 8.0 statistical software (GraphPad Prism, RRID: SCR_002798) version #8, La Jolla, CA, USA) (34). Normality of distribution was assessed using the D’Agostino Pearson normality test. MiR-197-3p expression, colony intensity, target mRNA expression levels and percentage of wound closure did not follow a normal distribution. Therefore, the non-parametric Kruskal–Wallis, 95% confidence interval (CI) followed by Dunn’s multiple comparisons test, was applied (42, 43). In contrast, western blot band intensity, optical density (OD) value and frequence of apoptotic cells (parent %) were normally distributed and were analyzed using one-way analysis of variance (ANOVA) with Tukey’s multiple comparisons test. All experiments were performed using independent biological replicates (n=3). P-values <0.05 were considered statistically significant.

## 3 Results

### 3.1 MiR-197-3p is significantly overexpressed in HPM cells and can be effectively modulated by mimic and antagomiR treatments

**Figure 1:**
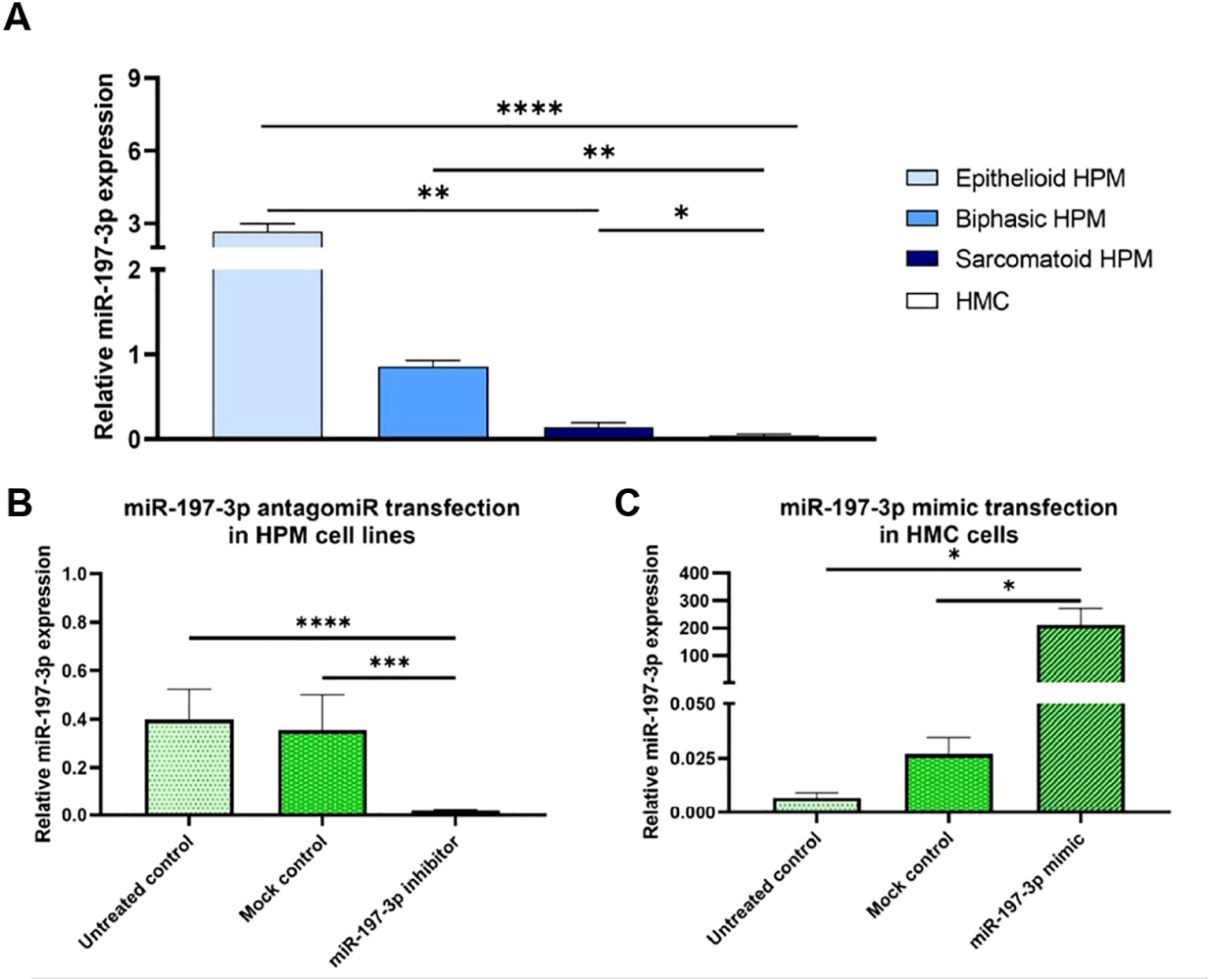
MiR-197-3p expression levels in HPM and HMC cells and effectiveness of miR-197-3p transfection. Intracellular miR-197-3p quantification was carried out by ddPCR and normalized to U6 snRNA. Results are shown as mean ± standard deviation of the mean (SD) of three replicates **(A)**. MiR-197-3p levels in HPM cells lines (epithelioid, biphasic and sarcomatoid HPM cell lines) transfected with miR-197-3p inhibitor **(B)**. MiR-197-3p levels in HMC cells transfected with miR-197-3p mimic **(C)**. Transfection efficacy was evaluated at 72h. Intracellular miR-197-3p quantification was normalized to U6 snRNA. Results are shown as mean ± standard deviation of the mean (SD) of three replicates. Statistical significance values: ****: p<0.0001; **: p<0.005; *: p<0.05; ***: p<0.001.

MiR-197-3p expression was analyzed at the intracellular level in HPM cells representing the epithelioid, biphasic, and sarcomatoid histotypes, as well as, in human mesothelial cells (HMC), used as control (**Figure 1A**). The results showed a significant upregulation of miR-197-3p in HPM compared to HMC cells (epithelioid vs. HMC ****p<0.0001; biphasic vs. HMC **p=0.0017 and sarcomatoid vs HMC *p=0.0228, respectively). Furthermore, epithelioid HPM cells exhibited a statistically higher miR-197-3p expression than sarcomatoid cells (**p=0.0012). Functional modulation further confirmed these expression patterns: antagomiR treatment of HPM resulted in a significant down-regulation of miR-197-3p expression compared to controls (vs untreated control: ****p<0.0001; vs mock: ***p=0.0003) (**Figure 1B**), whereas miR-197-3p mimic-transfected HMC cells showed a significant overexpression relative to both untreated (*p=0.045) and mock (*p=0.049) controls (**Figure 1C**).

### 3.2 MiR-197-3p knockdown reduces HPM cell proliferation activity without affecting their viability, migration and apoptosis

Given the altered expression of miR-197-3p detected in HPM cells, we investigated its functional role in these tumor cells. Since dysregulated miRNAs are frequently implicated in the regulation of cancer cell proliferation, we examined whether miR-197-3p influences the proliferative capacity of human pleural mesothelioma cells. To this aim, we performed a colony-forming assay to assess the grow capacity of HPM and HMC cells transfected with miR-197-3p inhibitor and mimic, respectively. AntagomiR transfection significantly inhibited colony formation compared to the untransfected control across all mesothelioma histotypes analyzed (epithelioid: *p=0.0240; biphasic: **p=0.0098; sarcomatoid: *p=0.0181). This decrease corresponded to a reduction of approximately 96% in epithelioid cells, 64% in biphasic cells, and 97% in sarcomatoid cells compared to controls.

**Figure 2:**
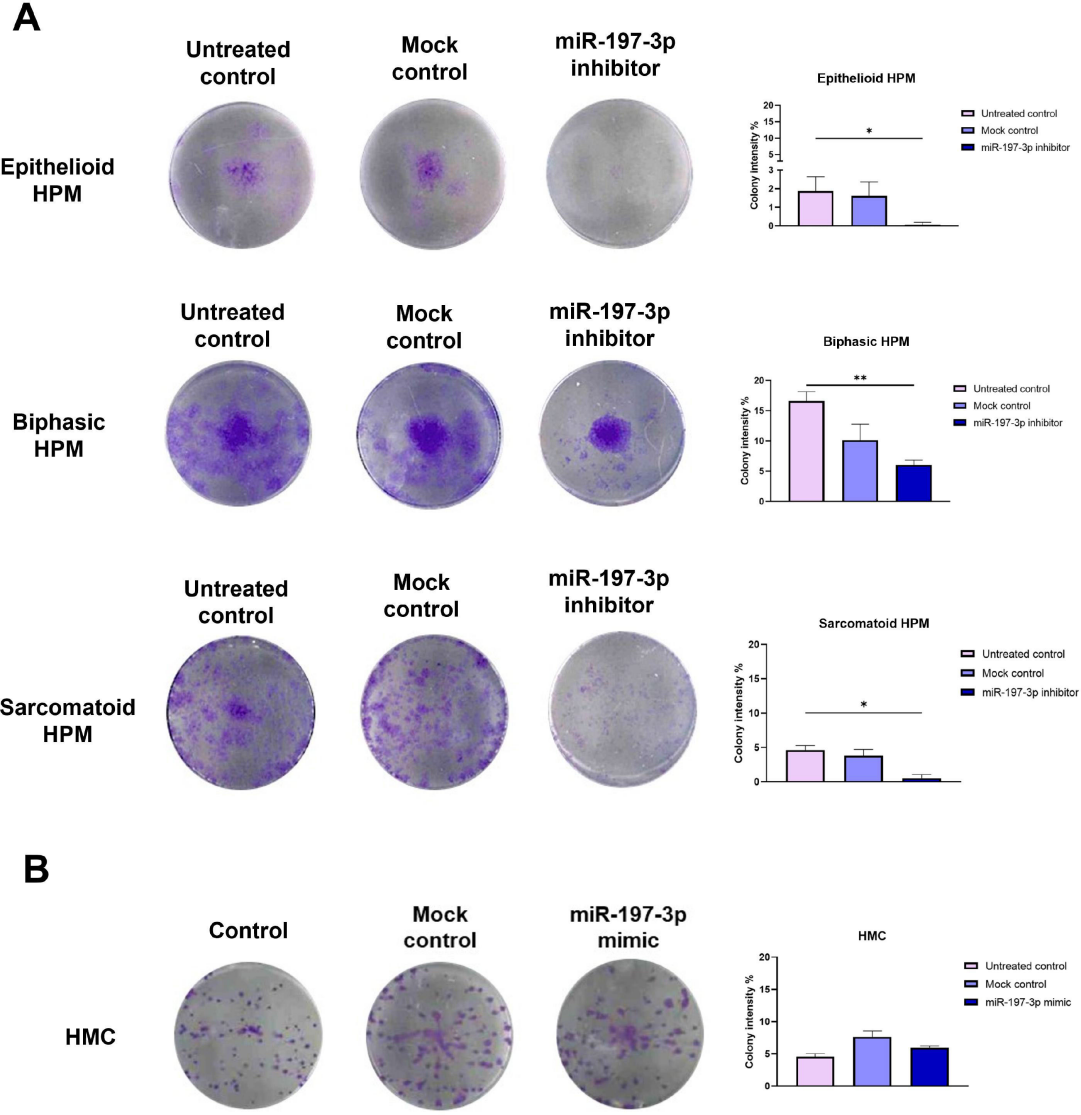
Colony formation assay. Analysis of cell clonogenic capacity was assessed by crystal violet. Staining was at day 10 after transfections with miR-197-3p inhibitor in HPM cell lines **(A)** and after mimic transfection in HMC cells **(B)**. Differences in the colony formation ability of transfected cells were evaluated by the assessment of colony intensity percent (%), quantified by ImageJ. Results are shown as mean ± standard deviation of the mean (SD) of three replicates. Statistical significance values: **: p<0.005; *: p<0.05.

Conversely, miR-197-3p mimic transfection in HMC cells did not result in a statistically significant increase in colony-forming ability (**Figure 2**).

Beyond its role in cell proliferation, we further explored the functional impact of miR-197-3p in HPM by evaluating additional cancer-related processes, including cell viability, migration, and apoptosis. Modulation of miR-197-3p expression through either antagomiR or mimic transfection did not produce significant changes in these parameters in HPM or HMC cells when compared to their respective controls (**Supplementary Figures 1** and **2**). These results suggest that miR-197-3p inhibition primarily affects clonogenic capacity without significantly impacting short-term cell viability or apoptosis under the experimental conditions tested.

### 3.3 Gene expression modulated by miR-197-3p in HPM cells

**Figure 3:**
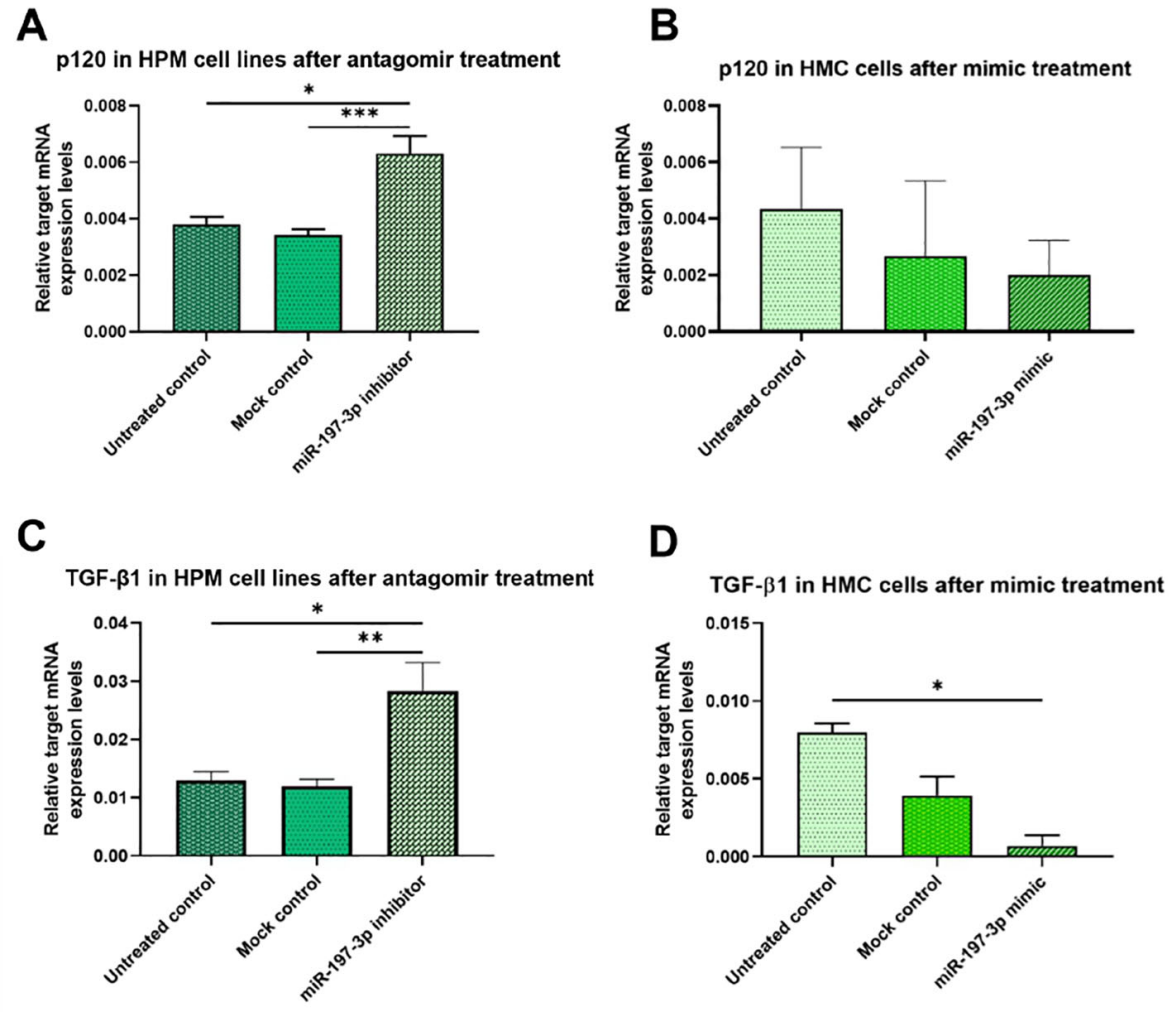
p120 and TGF-β1 mRNA expression following miR-197-3p gain- and loss-of-function experiments in HPM and HMC cells. Expression levels of p120 mRNA in HPM cell lines following miR-197-3p inhibitor transfection **(A)** and in HMC cells transfected with mimic miR-197-3p **(B)**, respectively. Expression levels of TGF-β1 mRNA in HPM cell lines following miR-197-3p inhibitor transfection **(C)** and in HMC cells transfected with mimic miR-197-3p **(D)**, respectively. Target expression was evaluated at 72h after transfection, by ddPCR. Target quantification was normalized to GAPDH. Results are shown as mean ± standard deviation of the mean (SD) of three replicates. Statistical significance values: ***: p<0.001; **: p<0.005; *: p<0.05.

To identify potential miR-197-3p targets involved in cell proliferation, we combined bioinformatic predictions with experimental validation using ddPCR and western blot analysis. Among the predicted candidates, catenin delta 1 (CTNND1/p120) emerged as a promising target. In HPM cells, p120 mRNA levels were significantly increased following miR-197-3p inhibition compared to control conditions (vs untreated control: *p=0.0280; vs mock: ***p=0.0003), corresponding to an approximately 1.6-fold increase (**Figure 3A**). In contrast, mimic-transfected HMCs showed a non-significant decrease in p120 mRNA expression (**Figure 3B**).

Although previous studies have reported an interaction between miR-197-3p and p120 (23), no significant changes in p120 protein levels were observed in either cell line, despite trends consistent with the observed mRNA expression patterns (**Supplementary Figure 3**).

A similar expression profile was observed for another predicted target, transforming growth factor beta 1 (TGF-β1). Specifically, compared to controls, TGF-β1 mRNA expression was significantly upregulated in inhibitor-transfected HPM cells (vs untreated control: *p=0.029; vs mock: **p=0.0063), corresponding to an approximately 2.2-fold increase (**Figure 3C**). Conversely, TGF-β1 expression was reduced in mimic-transfected HMCs (vs untreated control: *p=0.0328), showing an approximately 11-fold decrease compared to control levels (**Figure 3D**).

The impact of miR-197-3p on TGF-β1 protein levels was subsequently investigated by western blot. The analysis confirmed elevated TGF-β1 protein expression in inhibitor-transfected HPM cells — reaching statistical significance only when compared with untransfected controls — while mimic-transfected HMC cells exhibited a non-significant reduction (**Supplementary Figure 4**).

### 3.4 TGF-β1 and p120 3’ UTR contain potential miR-197-3p binding sites

**Figure 4:**
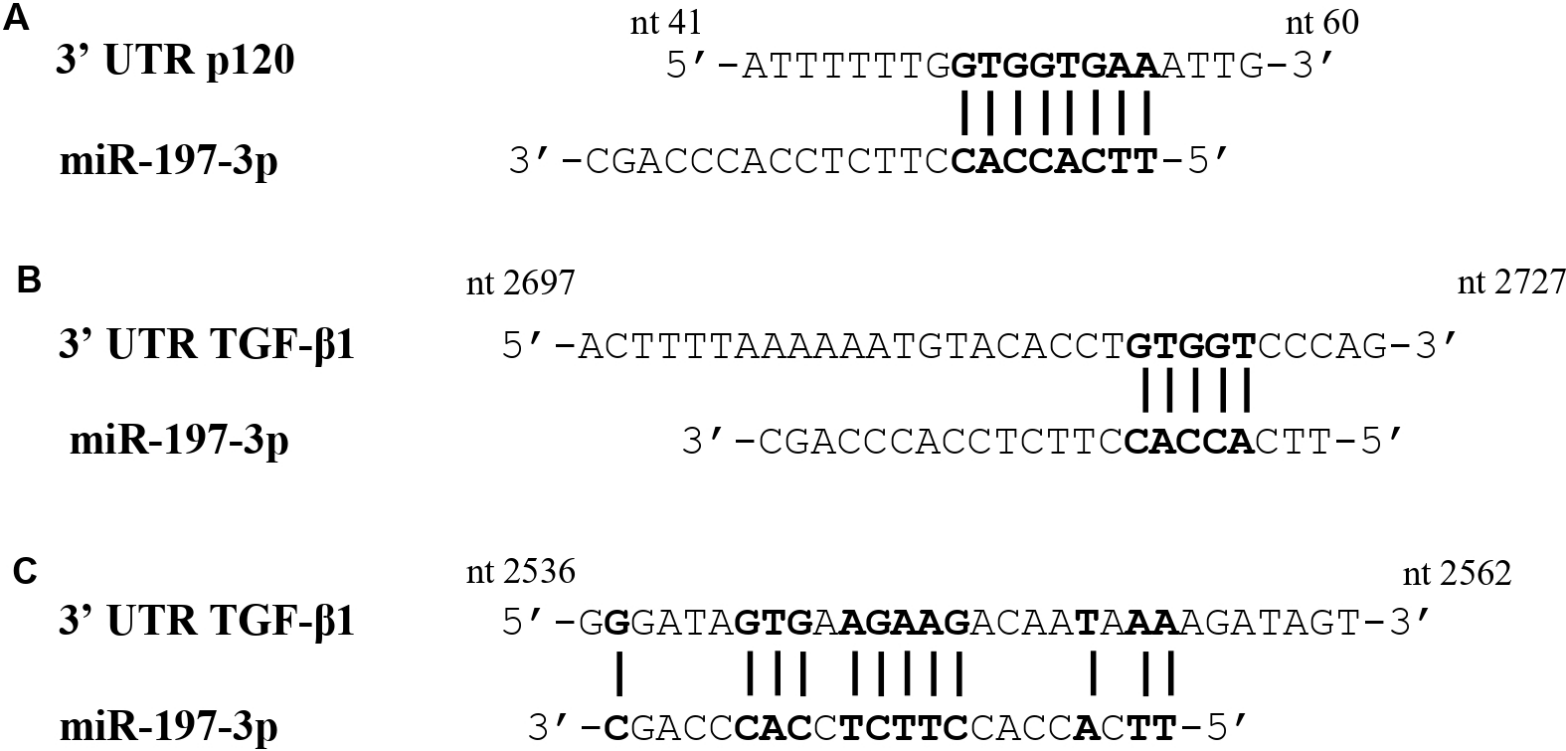
MiR-197-3p binding site on p120 mRNA and on TGF-β1 mRNA. Schematic representation of the interaction between p120 3′-UTR mRNA and miR-197-3p, showing the seed region of miR-197-3p with direct matching to the putative binding site on p120 mRNA **(A)**. Schematic representation of the interaction between TGF-β1 3′-UTR mRNA and miR-197-3p, showing the seed region of miR-197-3p with direct matching to the putative binding site on TGF-β1 mRNA **(B)** and the non-canonical binding site in the 3’ region of TGF-β1 mRNA **(C)**.

Bioinformatic analysis of the 3’-UTR sequences of p120 (NM_001085458) and TGF-β1 (NM_000660.7) revealed potential binding sites for miR-197-3p. Specifically, one site with perfect seed sequence complementarity was identified in the p120 3’-UTR (**Figure 4A**), while two sites were found in the TGF-β1 3’-UTR: one with complete seed match (**Figure 4B**) and another with partial complementarity involving the 3’ region of miR-197-3p (**Figure 4C**). These findings may explain the observed upregulation of p120 and TGF-β1 mRNAs in HPM cells following transfection with miR-197-3p antagomiRs.

## 4 Discussion

Human pleural mesothelioma (HPM) is a highly aggressive cancer, often diagnosed at advanced stages and lacking effective treatments (1, 2). In this context, microRNAs have emerged as key regulators of cancer-related pathways, offering valuable insights into tumor biology and response to therapies (44).

Given the critical role of epigenetic mechanisms in HPM, the present study investigated the expression and the functional relevance of miR-197-3p in key cancer-associated processes.

Herein, we report a significant up-regulation of miR-197-3p in HPM compared to HMC cells. Functional modulation experiments indicated that inhibition of miR-197-3p markedly reduced the clonogenic capacity of HPM cells across different histological subtypes. Conversely, miR-197-3p overexpression in HMC enhanced proliferative capacity, supporting a pro-proliferative role for this miRNA. These results are consistent with reports in other cancer types (22, 26, 27, 45), suggesting that miR-197-3p may be involved in conserved regulatory networks relevant to tumor growth.

To strengthen our findings, we analyzed three distinct HPM cell lines representing the main histological subtypes, along with HMC. Efficient modulation of miR-197-3p expression was obtained with both mimic and antagomiR molecules. Specifically, antagomiR treatment successfully suppressed miR-197-3p levels, consistent with the known mechanism of miRNA silencing through target degradation (46).

Since we observed a significant decrease in clonogenic growth following miR-197-3p inhibition, we investigated whether this effect was accompanied by changes in other cellular processes involved in HPM progression. Interestingly, modulation of miR-197-3p did not significantly alter cell viability, migration, or apoptosis. This apparent discrepancy can be explained by the distinct biological processes measured by the different assays employed. Specifically, colony formation reflects long-term proliferative and clonogenic potential, whereas the CCK-8 assay primarily measures short-term metabolic activity, and apoptosis assays assess cell death. Therefore, the lack of changes in short-term viability and apoptosis does not contradict the reduction in clonogenic capacity observed following miR-197-3p. Taken together, these findings suggest that miR-197-3p may preferentially affect long-term proliferative potential of HPM rather than their short-term metabolic activity. Certainly, cell cycle analysis would be useful to better define this aspect. These observations raise the possibility that miR-197-3p drives HPM cells toward a quiescent-like state or activates compensatory mechanisms, such as autophagy or stress responses (47), which may help to maintain the cellular homeostasis in the face of reduced proliferative signaling.

This interpretation is supported by previous studies reporting that miR-197-3p can regulate proliferation independently of apoptosis, potentially through modulation of cell cycle–associated pathways rather than caspase-dependent mechanisms (19). Furthermore, the frequent occurrence of p53 alterations in subsets of HPM may further uncouple proliferative control from apoptotic signaling, allowing cells to tolerate reduced growth without undergoing cell death (19). Consistently, mesothelioma cells might activate adaptive mechanisms, including autophagy, to maintain viability despite reduced proliferation (47). For example, in nasopharyngeal carcinoma, miR-197-3p regulates autophagy via HSPA5, which can support cell survival (47).

To gain insight into potential molecular mediators underlying these effects, we explored predicted miR-197-3p targets associated with proliferation, focusing on CTNND1 (p120-catenin) and TGF-β1. Both molecules have context-dependent roles in regulating proliferation (48, 49). TGF-β1, in particular, is known to have dual roles in cell proliferation, acting as a tumor suppressor by inducing cell cycle arrest and promoting differentiation, in early tumorigenesis, while promoting tumor progression in later stages, depending on the cellular context and signaling intensity (50) Similarly, p120-catenin has been reported to suppress proliferation and tumor growth in certain cancers, such as oral squamous cell carcinoma (51) suggesting a potential tumor-suppressive function in specific contexts. Our results suggest that miR-197-3p overexpression in HPM may suppress the expression of both TGF-β1 and p120, potentially relieving their inhibitory influence on proliferation and contributing to disease progression.

Our data, from gain- and loss-of-function experiments, supported a regulatory interaction between miR-197-3p and p120 mRNA, as antagomiR treatment significantly increased p120 mRNA levels. However, this increase was not mirrored at the protein level, suggesting the involvement of post-transcriptional regulatory mechanisms or protein turnover. In contrast, miR-197-3p inhibition resulted in elevated TGF-β1 expression (52, 53). Through a detailed bioinformatic analysis, we identified one site in the 3’-UTR of p120 and two distinct regions in the 3’-UTR of TGF-β1 mRNA complementary to the miR-197-3p seed sequence. These target sites may contribute to the regulatory interactions involving miR-197-3p. Notably, the relationship between miR-197-3p and TGF-β1 signaling has been reported in other cancer contexts (40) supporting the existence of conserved regulatory circuits. TGF-β1 is a multifunctional cytokine, with significant roles in distinct cell processes, such as growth, differentiation, immune regulation, and tissue repair, but its role in cancer remains complex and context-dependent (48, 54). Although our data suggest a potential regulatory association between miR-197-3p and the targets investigated, the mechanistic evidence provided in this study remains preliminary. In particular, the modulation observed at the mRNA level was not consistently reflected at the protein level, especially in the case of p120, and only partially for TGF-β1. Moreover, direct binding between miR-197-3p and the 3′-UTR of these targets was not experimentally validated, and functional rescue experiments were not performed. Therefore, the proposed regulatory interactions should be considered putative and require further investigation to establish a causal relationship.

In addition to these mechanistic considerations, other limitations should be acknowledged. In particular, our study relies on established HPM cell lines, with only one cell line representative of each of the three major histological subtypes included. Although these models allowed us to capture subtype-specific features, they may not fully reflect the biological heterogeneity of human pleural mesothelioma. In this regard, the use of additional mesothelioma cell lines, as well as more physiologically relevant systems such as patient-derived primary cultures or patient-derived xenograft (PDX) models, would be valuable to further support and extend our findings. Nevertheless, preliminary data obtained from primary HPM cultures are consistent with our results, supporting the potential relevance of miR-197-3p in more physiologically representative contexts. Additional studies using a broader range of experimental models will be required to better define the biological significance of these observations.

In conclusion, our results indicate that miR-197-3p is upregulated in HPM cells and may be associated with enhanced clonogenic capacity, potentially through modulation of proliferation-related pathways. While these findings suggest that miR-197-3p may be implicated in pathways relevant to HPM pathobiology, its precise mechanistic contribution and functional significance remain to be fully defined. Further studies are needed to clarify its role within mesothelioma-associated molecular networks and to evaluate miR-197-3p as a candidate for further investigation in HPM.

## Data Availability

The raw data supporting the conclusions of this article will be made available by the authors, without undue reservation.

## Supplementary material

Supplementary Figure 1 Cell viability assay and wound-healing assay. **(A)** CCK8 absorbance in epithelioid, biphasic, sarcomatoid HPM cell lines transfected with miR-197-3p inhibitor and HMC cells transfected with mimic. Cell viability was evaluated at 24h, 48h, and 72h after transfection. The results are expressed as mean of normalized OD values at 450 nm ± standard deviation of the mean (SD) of three replicates. At 24h and 48h epithelioid HPM showed a statistically significant decrease in viability following antagomiR transfection (*p ≤ 0.05; **p ≤ 0.01; ***p ≤ 0.001), while only biphasic HPM showed a significant decrease in cellular viability at 72h after transfection. **(B)** Analysis of cell migration ability, by scratch test, at 0 and 24 h after transfections with miR-197-3p inhibitor in HPM cell lines and mimic in HMC cells. Differences in cell migration of transfected cells were calculated according to the following formula: wound healing percentage = [(At0- Atx)]/At0 x 100%, quantified by ImageJ, and presented as the percentage (%) of wound closure ± standard deviation of the mean (SD) of three replicates.

Supplementary Figure 2 Flow cytometry analysis of apoptosis rates in HPM cells following miR-197-3p antagomiR and mimic transfection. Apoptosis rates in HPM cells transfected with miR-197-3p inhibitor (p>0.05). The analysis was carried out after 72 h of transfection. Apoptosis rates were depicted both as representative flow cytometry dot plots **(A)** and as apoptosis histograms **(B)**. Apoptosis rates in HMC cells transfected with mimic-miR-197-3p (p>0.05). The analysis was carried out after 72 h of transfection. Apoptosis rates were depicted both as representative flow cytometry dot plots **(C)** and as apoptosis histogram **(D)**. The results are expressed as the mean percentage (%) of apoptotic cells ± standard deviation of the mean (SD) of three replicates.

Supplementary Figure 3 p120 protein expression following miR-197-3p gain- and loss-of-function experiments in HPM and HMC cells. Effects of miR-197-3p on p120 protein were evaluated by western blot analysis using a selective antibody against p120 (110 KDa) 72 h after transfection with miR-197-3p inhibitor in HPM cell lines **(A)** and mimic transfection in HMC cells **(B)**. Differences in p120 protein expression were evaluated by the densitometry quantification of protein levels and normalized to β-actin (44 KDa). Results are shown as mean ± standard deviation of the mean (SD) of three replicates.

Supplementary Figure 4 Effects of gain and loss of function experiments on TGF-β1 protein expression level. The influence of miR-197-3p on TGF-β1 protein was investigated by western blot analysis using a selective antibody against TGF-β1 (43–55 KDa), at 72h after transfections with miR-197-3p inhibitor in HPM cell lines **(A)** and mimic transfection in HMC cells **(B)**. Differences in TGF-β1 protein expression were tested by the densitometry quantification of protein levels, normalized to β-actin (44 KDa). Results are shown as mean ± standard deviation of the mean (SD) of three replicates.
