# Supplementary material for: Proliferation of pleural mesothelioma cells is enhanced by the microRNA-197-3p activity
Source: Front Oncol. 2026 Jun 29;16:1807528. doi: 10.3389/fonc.2026.1807528 (PMC13357976; doi:10.3389/fonc.2026.1807528)
Supplement: Supplementary Figure 1 — Cell viability assay and wound-healing assay. (A) CCK8 absorbance in epithelioid, biphasic, sarcomatoid HPM cell lines transfected with miR-197-3p inhibitor and HMC cells transfected with mimic. Cell viability was evaluated at 24h, 48h, and 72h after transfection. The results are expressed as mean of normalized OD values at 450 nm ± standard deviation of the mean (SD) of three replicates. At 24h and 48h epithelioid HPM showed a statistically significant decrease in viability following antagomiR transfection (*p ≤ 0.05; **p ≤ 0.01; ***p ≤ 0.001), while only biphasic HPM showed a significant decrease in cellular viability at 72h after transfection. (B) Analysis of cell migration ability, by scratch test, at 0 and 24 h after transfections with miR-197-3p inhibitor in HPM cell lines and mimic in HMC cells. Differences in cell migration of transfected cells were calculated according to the following formula: wound healing percentage = [(At0- Atx)]/At0 x 100%, quantified by ImageJ, and presented as the percentage (%) of wound closure ± standard deviation of the mean (SD) of three replicates. [file DataSheet1.pdf]

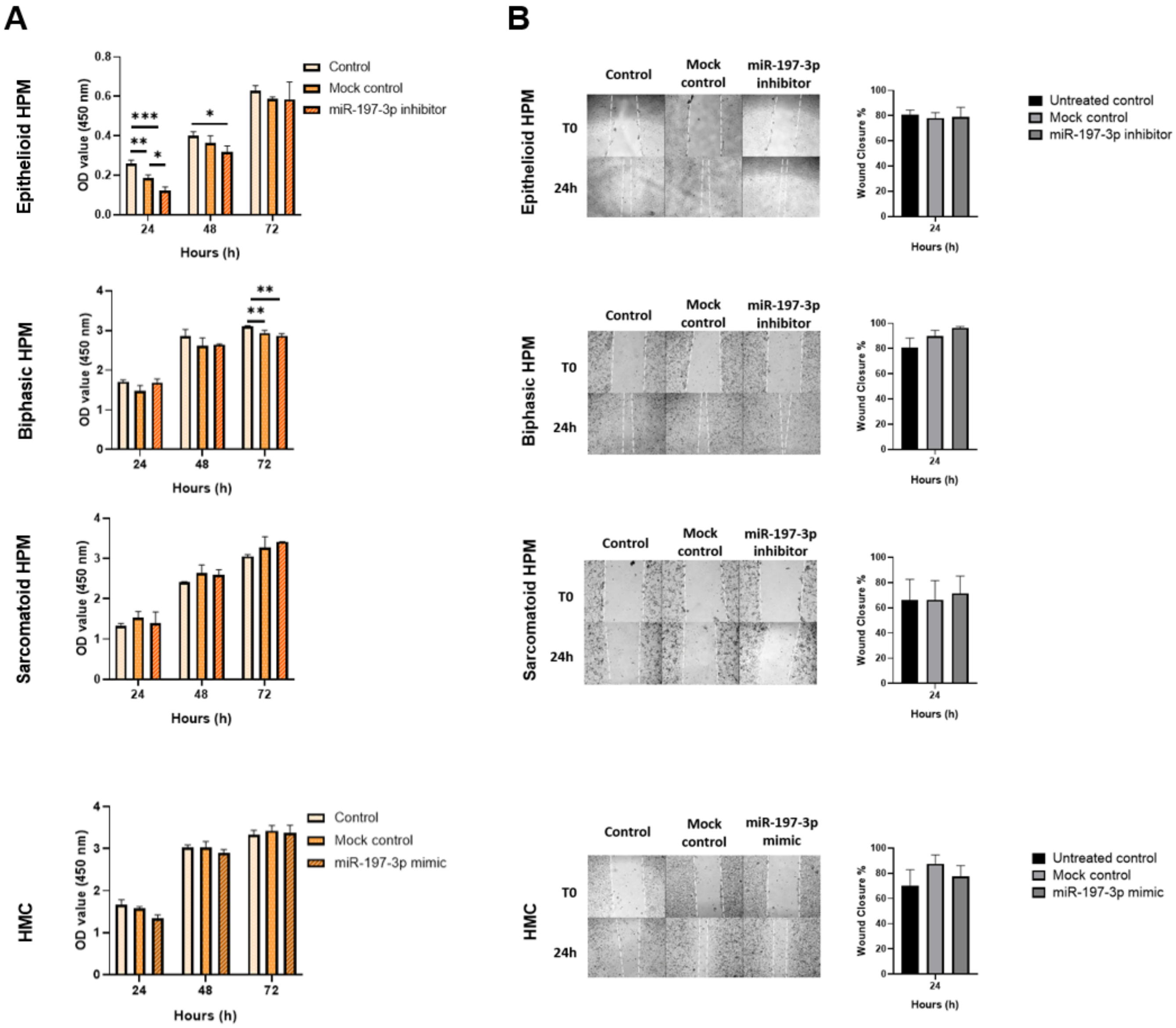
A
B
Epithelioid HPM
Biphasic HPM
Sarcomatoid HPM
HMC
OD value (450 nm)
Hours (h)
Control
Mock control
miR-197-3p inhibitor
miR-197-3p mimic
Mock
control
miR-197-3p
inhibitor
mimic
T0
24h
Wound Closure %
Untreated control
