# Supplementary figures and images for: Proliferation of pleural mesothelioma cells is enhanced by the microRNA-197-3p activity
Source: Front Oncol. 2026 Jun 29;16:1807528. doi: 10.3389/fonc.2026.1807528 (PMC13357976; doi:10.3389/fonc.2026.1807528)

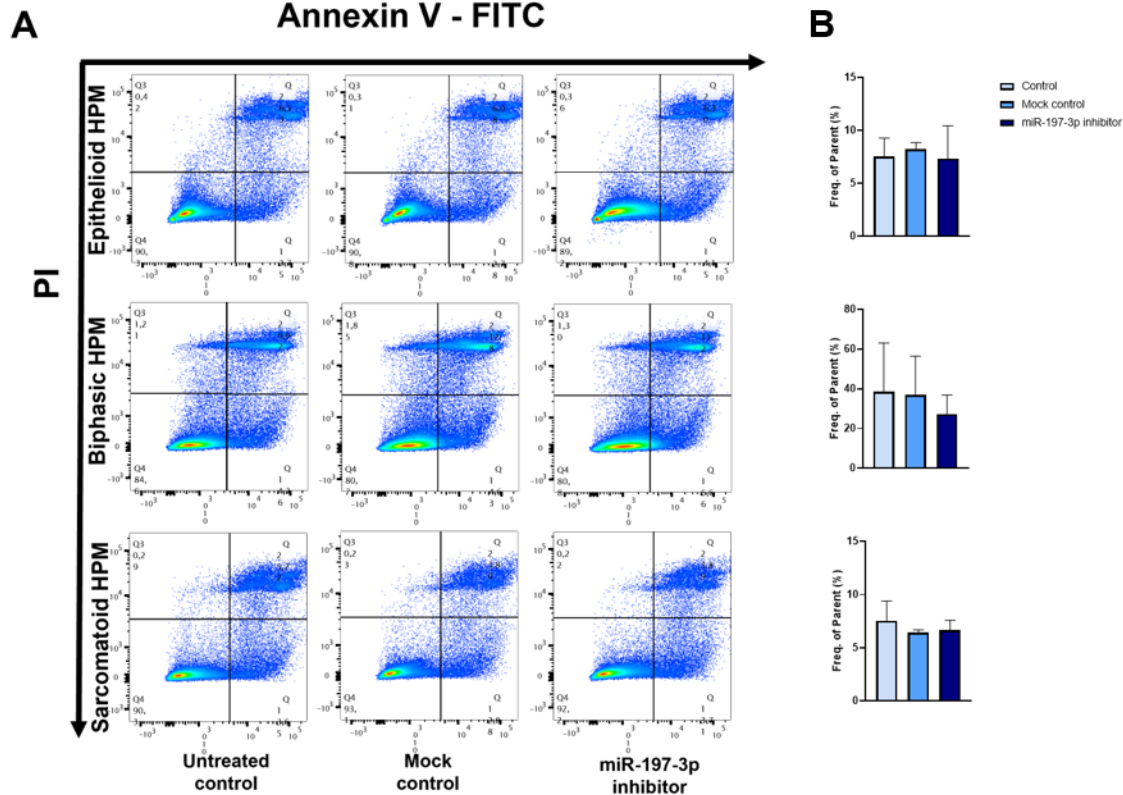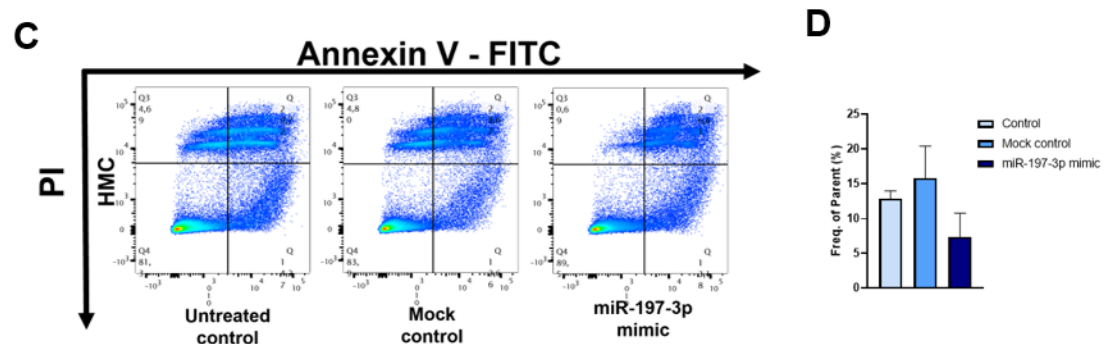

Supplement: Supplementary Figure 2 — Flow cytometry analysis of apoptosis rates in HPM cells following miR-197-3p antagomiR and mimic transfection. Apoptosis rates in HPM cells transfected with miR-197-3p inhibitor (p>0.05). The analysis was carried out after 72 h of transfection. Apoptosis rates were depicted both as representative flow cytometry dot plots (A) and as apoptosis histograms (B). Apoptosis rates in HMC cells transfected with mimic-miR-197-3p (p>0.05). The analysis was carried out after 72 h of transfection. Apoptosis rates were depicted both as representative flow cytometry dot plots (C) and as apoptosis histogram (D). The results are expressed as the mean percentage (%) of apoptotic cells ± standard deviation of the mean (SD) of three replicates. [file DataSheet2.pdf]

**A**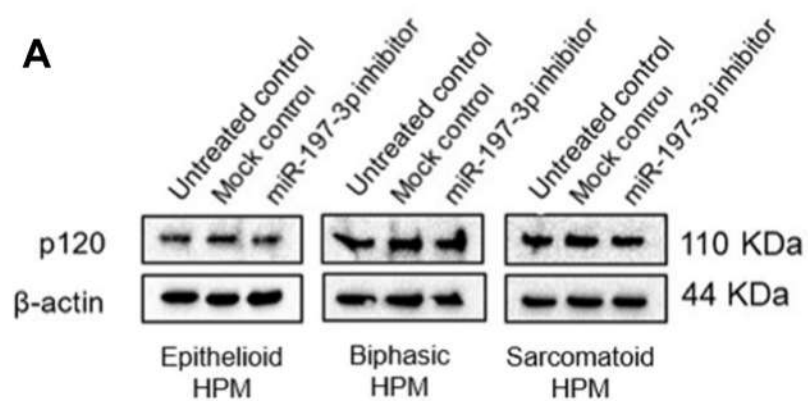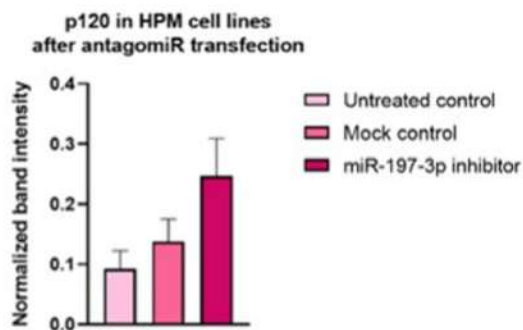**B**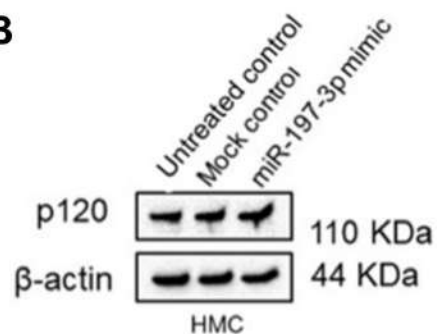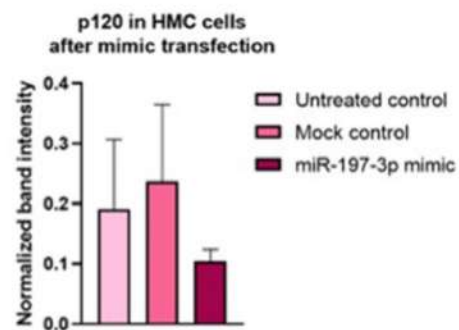

Supplement: Supplementary Figure 3 — p120 protein expression following miR-197-3p gain- and loss-of-function experiments in HPM and HMC cells. Effects of miR-197-3p on p120 protein were evaluated by western blot analysis using a selective antibody against p120 (110 KDa) 72 h after transfection with miR-197-3p inhibitor in HPM cell lines (A) and mimic transfection in HMC cells (B). Differences in p120 protein expression were evaluated by the densitometry quantification of protein levels and normalized to β-actin (44 KDa). Results are shown as mean ± standard deviation of the mean (SD) of three replicates. [file DataSheet3.pdf]

**A**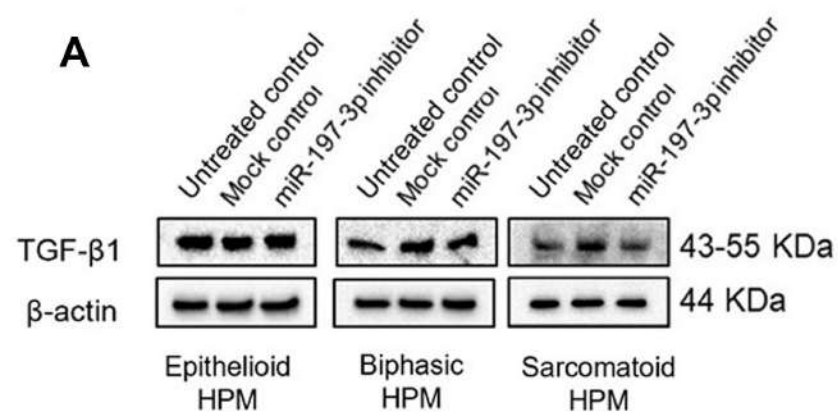

TGF-β1 in HPM cells  
after antagomiR transfection

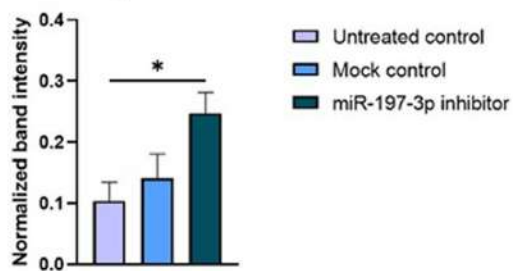**B**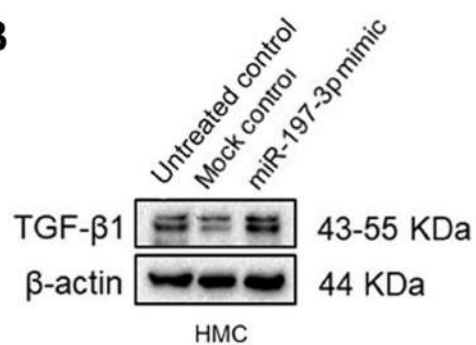

TGF-β1 in HMC cells  
after mimic transfection

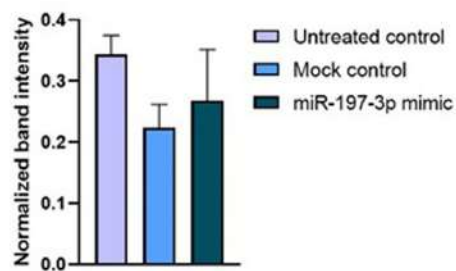

Supplement: Supplementary Figure 4 — Effects of gain and loss of function experiments on TGF-β1 protein expression level. The influence of miR-197-3p on TGF-β1 protein was investigated by western blot analysis using a selective antibody against TGF-β1 (43–55 KDa), at 72h after transfections with miR-197-3p inhibitor in HPM cell lines (A) and mimic transfection in HMC cells (B). Differences in TGF-β1 protein expression were tested by the densitometry quantification of protein levels, normalized to β-actin (44 KDa). Results are shown as mean ± standard deviation of the mean (SD) of three replicates. [file DataSheet4.pdf]
